# DNA Methylation in Healthy Older Adults With a History of Childhood Adversity—Findings From the Women 40+ Healthy Aging Study

**DOI:** 10.3389/fpsyt.2019.00777

**Published:** 2019-10-23

**Authors:** Serena Fiacco, Elena Silvia Gardini, Laura Mernone, Lea Schick, Ulrike Ehlert

**Affiliations:** ^1^Clinical Psychology and Psychotherapy, University of Zurich, Zurich, Switzerland; ^2^URPP Dynamics of Healthy Aging Research Priority Program, University of Zurich, Zurich, Switzerland

**Keywords:** early life adversity, healthy women, methylation, NR3C1, ERα, cortisol, estradiol

## Abstract

**Background:** Adversity in early development seems to increase the risk of stress-related somatic disorders later in life. Physiologically, functioning of the hypothalamic–pituitary–adrenal and hypothalamic–pituitary–gonadal axes is often discussed as long-term mediators of risk. In particular, DNA methylation in the glucocorticoid receptor gene promoter (*NR3C1*) has been associated with type and strength of early life adversity and subsequent effects on HPA axis signaling in humans. Animal studies, moreover, suggest changes in DNA methylation in the estrogen receptor gene (*ER*α) upon early life adversity. We investigated the association of type and severity of childhood adversity with methylation in *NR3C1* and *ER*α and additionally considered associations between methylation and steroid hormone secretion.

**Methods:** The percentage of methylation within the *NR3C1* promoter and the *ER*α shore was investigated using dried blood spot samples of 103 healthy women aged 40–73 years. Childhood adversity was examined with the Childhood Trauma Questionnaire. Linear regression analyses were performed with methylation as dependent variable and the experience of emotional abuse and neglect, physical abuse and neglect, and sexual abuse (compared to non-experience) as independent variables. All analyses were controlled for age, BMI, annual household income, and smoking status and were adjusted for multiple testing.

**Results:** Overall, over 70% of the sample reported having experienced any kind of abuse or neglect of at least low intensity. There were no significant associations between childhood adversity and methylation in the *NR3C1* promoter (all *p* > .10). Participants reporting emotional abuse showed significantly higher methylation in the *ER*α shore than those who did not (*p* = .001). Additionally, higher levels of adversity were associated with higher levels of *ER*α shore methylation (*p* = .001).

**Conclusion:** In healthy women, early life adversity does not seem to result in NR3C1 promoter hypermethylation in midlife and older age. This is the first study in humans to suggest that childhood adversity might, however, epigenetically modify the ERα shore. Further studies are needed to gain a better understanding of why some individuals remain healthy and others develop psychopathologies in the face of childhood adversity.

## Introduction

Individuals with a history of abuse or neglect in early life are at risk of developing psychopathology later in life (1, 2). Such adverse experiences can have long-lasting effects on the physiological adaptation to stress and thus increase the risk of depression (3, 4), stress-related somatic disorders such as somatoform pain disorder and fibromyalgia (5), or posttraumatic stress disorder (PTSD; 6). Indeed, Kessler et al. (7) estimated that nearly 30% of all psychiatric disorders across countries can be traced back to childhood adversity. As women are generally at a higher risk of stress-related disorders than men (8, 9), women with a history of childhood adversity might represent an especially vulnerable group of individuals (10).

Early life stress can lead to a disruption in stress-sensitive systems such as the hypothalamic–pituitary–adrenal (HPA) axis. This axis is activated by acute stress and responds with a release of adrenal glucocorticoids such as cortisol into the bloodstream (11). Cortisol then exerts both enhancing and suppressing effects, depending on the target tissue (12). Once the stressor is withdrawn, the HPA axis down-regulates its own activity through a negative feedback loop, which is mainly mediated by binding of circulating cortisol molecules to glucocorticoid receptors (*GR*) in the hippocampus, hypothalamus, and the amygdala (13–15). While the human body is prepared to adapt upon acute stress, repeated or extreme stress exposure with insufficient or inaccurate adaptation can be damaging to health (16). Early life represents a sensitive time during which stress exposure can have long-lasting effects on the future stress adaptation (17). Previous studies found that individuals who had experienced childhood adversity showed a characteristic cortisol stress response. Specifically, moderate to severe childhood trauma and childhood emotional abuse were associated with a lower cortisol release in response to the dexamethasone-/corticotropin-releasing hormone (DEX/CRH) test (18, 19). Moreover, childhood physical abuse was associated with a blunted cortisol response to the Trier Social Stress Test (TSST), when compared to women without physical abuse (20). These findings suggest first a characteristic change in HPA axis signaling, which might result from different types of trauma and/or from a dose-response relationship, as different types of adversity regularly co-occur. Second, these effects were found not only in clinical samples but also in non-clinical samples and therefore suggest an effect of early life adversity on the HPA axis, which is independent of current psychopathology.

Among other mechanisms, early life adversity may lead to epigenetic DNA modifications in genes related to HPA axis signaling in order to promote adaptation to possible adversity later in life (21, 22 for reviews). Epigenetic DNA modifications can influence gene expression and behavior over a long period of time and therefore critically influence the health status of the entire organism (23). Methylation is thought to be the most stable epigenetic DNA modification and has therefore been studied in the context of the long-term effects of early life adversity on health and disease (24, 25).

Human studies suggest a causal role of early life adversity in methylation of the gene encoding the *GR* (mainly exon 1_F_ of the *NR3C1* gene promoter; 21, 26, 27). Higher methylation in hippocampal *NR3C1* lowers this specific gene expression and reduces the number of *GR*s. This leads to a diminished *GR*-mediated negative feedback loop and, in turn, to an exaggerated glucocorticoid secretion (28, 29). Postmortem analyses allow a direct investigation of hippocampal *NR3C1* methylation in humans. In a rare study in suicide victims, those who had experienced childhood abuse showed higher hippocampal *NR3C1* promoter methylation and a decreased *GR*1_F_ expression compared to those without childhood abuse (30). It is difficult to disentangle the effects of early life adversity and current or past psychiatric disorder on methylation patterns, as disorders can themselves have effects on biomarkers (31). Therefore, the few studies in healthy adults with a history of childhood adversity are especially valuable for detecting whether methylation in *NR3C1* is responsive to early life adversity. In such adults, greater childhood adversity was associated with higher methylation in *NR3C1* (32, 33). These findings suggest that early life adversity might pose a significant independent risk factor for *NR3C1* methylation. Nevertheless, it should be mentioned that recent studies were unable to replicate this association (34, 35).

Possibly, *NR3C1* methylation affects HPA axis signaling in women to a greater extent than in men. First results show that, in otherwise healthy women, higher methylation in *NR3C1* was associated with higher cortisol secretion provoked by the TSST. In healthy men, by contrast, the *NR3C1* methylation and cortisol response were not associated (36). In line with these findings, healthy at high-risk men and women from the Dutch Famine Birth Cohort showed higher cortisol secretion in response to a psychological stress test, with higher methylation in *NR3C1*. The effect disappeared when controlling for sex (37). Circulating estradiol (E2) levels might mediate the association between *NR3C1* methylation and HPA axis response. As E2 levels are generally higher in women than in men, this would explain why women’s HPA axis response is more sensitive to *NR3C1* methylation. More precisely, E2 takes effect upon binding to estrogen receptors (*ER*s). Two distinct forms of intracellular receptors mediate genomic effects of circulating estrogens: estrogen receptor alpha (*ER*α) and estrogen receptor beta (*ER*β) (38). Additionally, the G protein-coupled estrogen receptor (*GPER*) mediates rapid non-genomic effects of estrogens (39). *ER*s are abundantly expressed in cells of the hypothalamus, the pituitary, and the adrenal (39–41). All of these structures represent key players in HPA axis signaling, and E2 can therefore intervene with the negative feedback regulation of the HPA axis (reviewed in 42, 43, 44). Women accordingly show a slightly different stress response than do men (45, 46), which may be one of the reasons for women’s pronounced risk of stress-related disorders (4, 47).

Animal studies suggest that the early social environment can influence *ER*α expression (48). In animal models, mothers represent the main source of social attachment. Early maternal care is assumed to influence the infant’s *ER*α expression through its effects on DNA methylation in the *ER*α gene. Female rat offspring that received low maternal care in the form of licking and grooming (LG) were found to have a higher average methylation in the exon 1b *ER*α promoter region and lower *ER*α mRNA expression as adults than the offspring of high LG mothers (49, 50). It is suggested that, in particular, the imprint of low maternal care should prepare the infant for a future hostile environment (51). Maternal care and *ER*β are assumed to be independent (50), while *GPER* has never been investigated in the context of maternal care. To conclude, similar to the effects on *NR3C1* methylation, negative early life experiences such as low maternal care can differentially modify methylation in the *ER*α gene in female animal models. To the best of our knowledge, it has never been investigated whether the early environment, and specifically the experience of early life adversity, may have a lasting effect on *ERα* gene methylation in women.

We hypothesized that women with a history of childhood adversity would show higher methylation in the *NR3C1* promoter and higher methylation in the *ER*α shore than women without such a history. Moreover, we hypothesized that the type of adversity would be differentially associated with methylation state and that greater adversity would be associated with higher methylation in both the *NR3C1* promoter and *ER*α shore. We expected that higher methylation in the *NR3C1* promoter would be associated with basal steroid hormone profiles, indicated by cortisol levels and the E2 to cortisol ratio (E2/C). Finally, from an exploratory perspective, we expected an association between *ER*α shore methylation and basal E2 levels and the E2/C ratio.

## Materials and Methods

### The Current Study

The Women 40+ Healthy Aging Study was conducted at the University of Zurich and targeted subjectively healthy community-dwelling women between the age of 40 and 75. The present analyses are part of this large cross-sectional project, and the recruitment procedures are described in detail elsewhere (52).

Women reporting any acute or chronic somatic disease or mental disorder, or receiving any psychotherapeutic or psychopharmacological treatment during the last 6 months, were not included in the study. Moreover, habitual drinkers (more than two standard units of alcohol per day) were not included. Additional exclusion criteria were pregnancy (in the last 6 months), premature menopause or a surgical menopausal status (removal of either both ovaries or the uterus), intake of hormonal medication (oral contraceptives or hormone therapy in the last 6 months), shift work, and a recent long-distance flight. These criteria were in a first step assessed in an online self-screening and in a second step additionally confirmed by a trained study member in a telephone screening.

### Study Procedures

Participants were invited to a weekday laboratory session at the University of Zurich between June 2017 and February 2018. Participants were asked to avoid any physical exercise for at least 24 h prior to the session and were instructed not to eat or drink (except for water) on the day of the session (53). All laboratory sessions were conducted one to one (one participant with one study member) and started at 7.45 a.m. with brief instructions. At 8.00 a.m., one saliva sample and several capillary blood spots were collected (54). On the day following the laboratory session, participants completed an online survey comprising validated psychological questionnaires. The procedures were controlled for menstrual cycle phase in women with menstrual bleedings (pre- and perimenopausal women), as defined by information on bleeding strength and patterns (55). Sample size calculations were performed using G*Power 3.1 (56). Specifically, calculations were based on F-tests using linear multiple regression analysis with a fixed model and investigating an R^2^ increase. Under the assumption of a relatively small effect size (f^2^ = ^.^15; 57), we decided to collect data of 100 participants, yielding a power of around 0.95 to test the proposed hypotheses. The nominal alpha level of 0.05 was adjusted to.029 to take into account the number of hypotheses tested and the correlation between the predictors. Of the 130 women who completed the entire study, nine were not eligible for the data analyses due to medication intake prior to the laboratory session. The final sample size was therefore appropriate for the planned analyses. The study was conducted in accordance with the recommendations of the Cantonal Ethics Committee (KEK) Zurich, which approved the protocol. All subjects gave written informed consent in accordance with the Declaration of Helsinki.

### Childhood Adversity

Childhood adversity was investigated with the German version of the Childhood Trauma Questionnaire (CTQ; 58). Using the CTQ, a sub-form distinction was made between adverse childhood experiences in the form of physical, emotional, and sexual abuse and adverse childhood experiences in the form of physical and emotional neglect. Additionally, a maltreatment score was calculated by summing up all individual categories of childhood adversity exceeding a critical threshold defined by Walker et al. (59). Critical thresholds were set as follows: physical abuse (8 points), emotional abuse (9 points), sexual abuse (6 points), physical neglect (8 points), and emotional neglect (10 points) (60). Higher maltreatment values indicate more exceeded thresholds, with values ranging from (0) *no threshold exceeded* to (5) *five thresholds exceeded*.

### Methylation

#### DNA Extraction

Cytosine methylation was assessed from dried blood spot (DBS) DNA samples, as previously described elsewhere (see, e.g., 61, 62, 63). The Qiagen QIAamp DNA Investigator Kit (Qiagen, Hombrechtikon, Switzerland) was used to extract genomic DNA from three punches of blood-soaked filter paper (each 3 mm in diameter). Punches were then eluted in 30 μl of RNase-free water, and the DNA concentration was assessed using the Qubit Fluorometer (Thermo Fischer Scientific, Reinach, Switzerland). A total range in DNA from 41 to 168 ng was detected.

#### NGS Library Preparation

First, we performed bisulfite conversion of DNA using the EZ 96-DNA Methylation-Gold Kit (Zymo Research, Luzern, Switzerland). DNA was eluted in 20 μl of RNase-free water. The sequences of interest (*ER*α shore of promoter C: (hg 38) chr6:151,805,523-151,805,822 and *GR* promoter: (hg 38) Chr5: 143404021-143404338 were amplified using the following primers: *ER*α—frw 5’-GTTTTTTGTGAGTAGATAGTAAGTT-3’ and rws: 5’-AAACCTACCCTACTAAATCAAAAAC-3,’ *GR*: frw 5’-TTG AAG TTT TTT TAG AGG G-3’ and rws 5’-AAT TTC TCC AAT TTC TTT TCT C-3’ with the following thermocycler conditions: 95°C for 3 min, [98°C for 20 s, 58°C (*ER*α); 60°C (*NR3C1*) for 15 s, 72°C for 15 s] x 40, and a final step with 72°C for 45 s. Forward and reverse primers included universal primer sequences CS1/CS2 (Fluidigm, California, USA) on 5.’ The generated amplicons were then purified using the E-Gels 2% size selection technology (Thermo Fisher Scientific, Reinach, Switzerland). Indexing with unique single barcode (Fluidigm, California, USA) was then performed through a second PCR [95°C, 3 min; (98°C, 20 s; 60°C, 15 s; 72°C, 15 s) x 10; 72°C 45 s] on the purified amplicons. Samples were pooled and diluted (10x). The two libraries (*ER*α and *NR3C1*) were quantified using the Agilent 2200 Tape Station Instrument (Santa Clara, CA, USA) with HS DNA 1000 reagents. The two libraries (2 nM each) were merged, and sequencing was performed on the Illumina MiSeq sequencer using the V3, 600 cycles kit.

#### Interrogation of CpG Sites in the Targeted Amplicon

**Figure 1:**
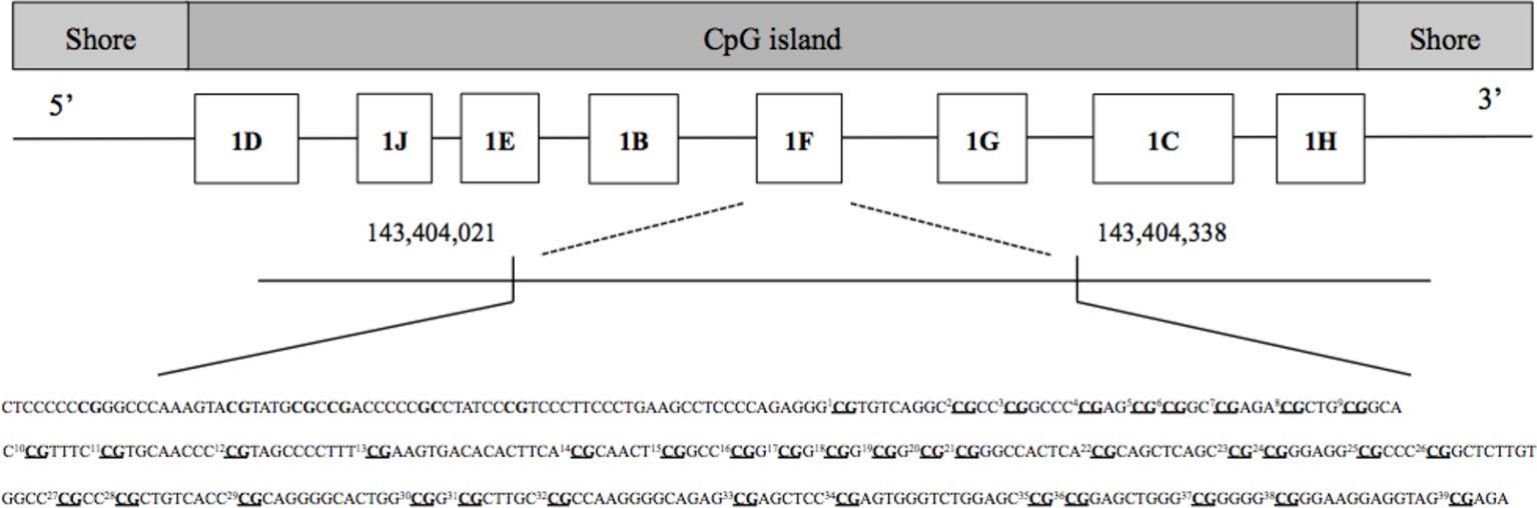
Investigated single CpG sites in the glucocorticoid receptor (*GR*) gene promoter (*NR3C1*). Underlined CpGs represent the 39 single targeted sites. For analysis, one mean % methylation score across all 39 investigated CpGs of interest was created to represent overall methylation in the *NR3C1* promoter region.

**Figure 2:**
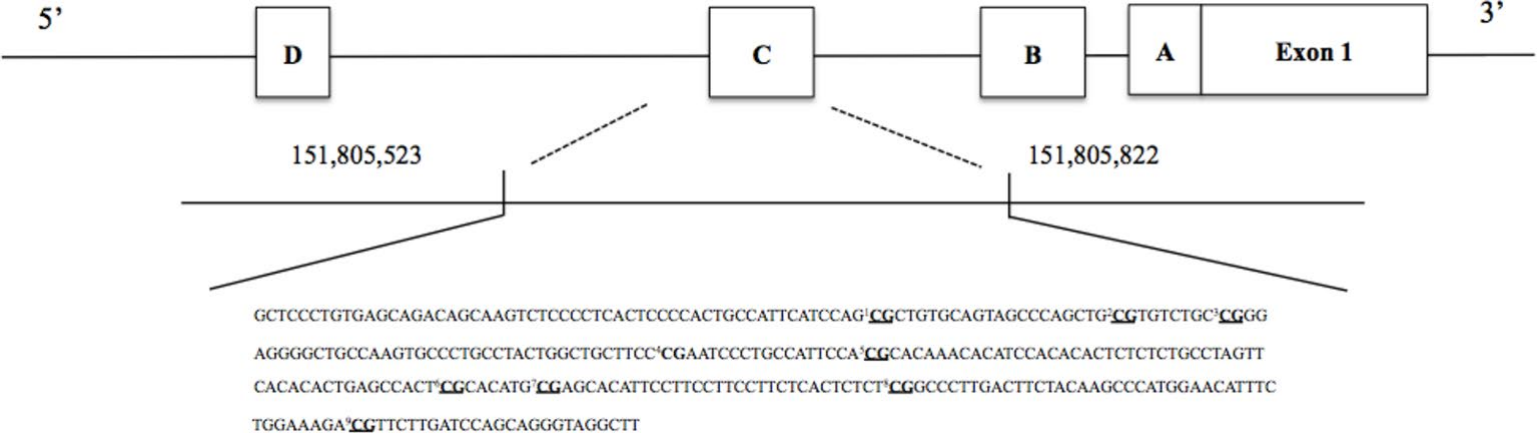
Investigated single CpG sites in the estrogen receptor alpha shore (*ER*α). Underlined CpGs represent the nine single targeted sites. To represent overall methylation in the *ER*α shore, one mean % methylation score across all nine investigated CpGs of interest was created.

Quality analysis of adaptor sequences and bases was performed using Trimmomatic v0.35 (64). Low-quality products were removed according to the default settings. Bismark software (v0.19.0) was used to extract the counts of methylated (cytosines) and unmethylated (thymine) bases. Unmethylated and methylated counts were summed up, and CpG sites scoring less than 100 counts were removed in line with Chen et al. (65). Finally, methylation percentages were calculated by dividing unconverted counts by the total number of counts (methylated and unmethylated). To represent overall methylation in the *NR3C1* promoter region, one mean % methylation score across all 39 investigated CpGs of interest was created. Single CpG sites are presented in **Figure 1** and were chosen in line with Palma-Gudiel et al. (21). A similar approach was applied to quantify methylation in the *ER*α shore of promoter C (66). Again, methylation across the nine investigated CpGs (see **Figure 2**) was represented by a mean % score across all sites. As previous studies have investigated the association of childhood adversity and single CpGs in the *NR3C1* promoter, these associations were also tested and the results can be found in the supplementary material.

### Steroid Hormone Levels

Saliva samples were assessed to analyze levels of E2 (pmol/L) and cortisol (nmol/L). All saliva samples were collected in 2-ml SaliCaps (IBL International GmbH, Hamburg, Germany) using the passive drool method. Saliva samples were stored at −20°C until biochemical analyses were performed. Thawed saliva samples were centrifuged and analyzed using enzyme-linked immunoassays (IBL International GmbH, Hamburg, Germany). Intra- and inter-assay variations were below 10%. Sensitivity was 1.10 pmol/L for E2 and 0.03 nmol/L for cortisol. The biochemical laboratory of the Department of Psychology, Clinical Psychology, and Psychotherapy at the University of Zurich performed the salivary analyses.

### Control Variables

Based on initial correlation analyses, all subsequent analyses were controlled for age, BMI, and socioeconomic status. Additionally, smoking status (in package-years) was controlled for due its known effect on methylation levels. Analyses involving steroid hormone levels were additionally controlled for the stress level in the week prior to the laboratory session, as assessed with the German version of the Perceived Stress Scale (PSS-10, 67).

### Statistical Analyses

First, based on age and menopausal stage, two E2 values (22.7; 25.75 pmol/L) were considered as implausibly high and therefore excluded from the analyses. Additionally, two cortisol values (morning level: 86.93; 8.00 a.m. level: 46.77 nmol/L) and three cases of *NR3C1* methylation (30.57; 38.06; 55.85%) were excluded from further analyses. This decision was based on the comparison of single values with information from previous studies (21, 68). For cortisol, reports of recent stressful experiences or lack of sleep, which may provide an explanation for considerably higher than average values, were additionally considered. Methylation data in the *NR3C1* promoter were highly left-skewed and therefore log-transformed to approach normal distribution. Additionally, steroid hormone data were log-transformed. There were missing CTQ data from 16 participants, who were therefore excluded from further analyses. To test the association between type of adversity and methylation, partial correlations between adversity sub-forms and methylation in the *NR3C1* promoter and the *ER*α shore were calculated. The association between strength of adversity and methylation was tested using partial correlations between the maltreatment score and methylation in the *NR3C1* promoter and the *ER*α shore. Finally, the association between methylation and steroid hormone levels was investigated using partial correlations. In an additional step, the results of the correlation analyses were verified using stepwise linear regression analyses including control variables (step one) and independent variables (step two). The analyses were adjusted for multiple testing. As proposed by Benjamini and Hochberg (69), the α-value of 0.05 was adjusted (multiplied) by (n+1)/2n (whereas n = 6). This adjustment was performed to account for the inter-dependence of the six CTQ sub-forms including the maltreatment score. Therefore, an α-value of *p* < 0.029 was considered statistically significant. For analyses regarding the association between methylation and steroid hormones, the level of statistical significance was set at *p* < .05. Analyses were performed using SPSS (version 23, IBL).

## Results

### Demographic Characteristics

**Table 1** summarizes the general sample characteristics of the entire study population. There were no differences between women with any kind of abuse or neglect and women without any such experience with regard to marital status (*p* = .74), education (*p* = .35), smoking status (*p* = .95), age (*p* = .14), and BMI (*p* = .16). Women without experience of childhood abuse had a slightly, although not significantly (*p* = .067), higher annual household income than those who had experienced such abuse. Moreover, women with a history of childhood adversity showed a significantly lower E2/C ratio than did women without such a history (*p* = .039; see **Table 2**).

**Table 1:** Descriptive statistics of the sample.

	N	%
*Country of origin*		
Switzerland	105	88.2
Germany, Austria, Liechtenstein	13	10.9
Hungary	1	0.9
*Marital status*		
Single	28	23.5
Married	63	52.9
Divorced	22	18.5
Widowed	6	5.0
*Education*		
Vocational education	42	35.3
High school-leaving certificate	23	19.3
College/University degree	53	44.5
Other	1	0.8
*Smoking*		
Yes	10	8.4
1–2 (cigarettes per day)	6	5.0
3–10 (cigarettes per day)	2	1.7
>10 (cigarettes per day)	2	1.7
	**Mean**	**SD**
*Type of adversity*		
Emotional abuse	8.64	4.11
Physical abuse	5.87	1.89
Sexual abuse	6.06	2.54
Emotional neglect	12.25	4.36
Physical neglect	7.58	2.96
Maltreatment score	1.99	1.69

**Table 2:** Descriptive statistics representing mean values of the total study sample and mean values based on participants’ history of childhood adversity.

	Total mean (SD)	With adversity mean (SD)	Without adversity mean (SD)	p-value
*Control variables*				
Age	53.37 (8.98)	54.00 (8.86)	51.00 (9.19)	.138
Annual household income (CHF)	127,874 (75, 505)	121,329 (71, 889)	152,479 (84, 884)	.067
Body mass index (kg/m^2^)	23.03 (3.96)	23.27 (3.82)	22.11 (3.01)	.163
Percent methylation				
*NR3C1 promoter*	.85 (2.78)	.77 (2.44)	1.11(3.72)	.60
*ERα shore*	77.11 (12.32)	77.78 (11.20)	74.44 (16.07)	.25
*Steroid hormones*				
Estradiol (pmol/L)	6.15 (5.22)	5.79 (5.00)	7.54 (5.84)	.137
Waking cortisol (nmol/L)	7.05 (10.40)	5.54 (4.87)	7.30 (6.52)	.145
08.00 a.m. cortisol (nmol/L)	6.64 (5.73)	6.37 (4.36)	6.05 (4.55)	.754
Estradiol/cortisol ratio	1.36 (1.43)	1.22 (1.56)	1.90 (2.13)	.039

CHF, Swiss Francs. Statistics represent two-tailed comparisons of unstandardized mean values between women with vs. without a history of childhood adversity.

### Prevalence of Childhood Adversities

The prevalence rates of the sub-forms of abuse and neglect are depicted in **Table 3**. Overall, 70.6% of the sample reported having experienced any kind of abuse or neglect of at least low intensity.

**Table 3:** Classification of traumatic childhood events with the Childhood Trauma Questionnaire (CTQ) sub-forms.

	None (or minimal)	Low (to moderate)	Moderate (to severe)	Severe (to extreme)	No information
Emotional abuse	82 (67.6%)	17 (13.9%)	12 (10.0%)	4 (3.4%)	6 (5.1%)
Physical abuse	103 (85.1%)	7 (5.7%)	4 (3.4%)	2 (1.7%)	5 (4.1%)
Sexual abuse	92 (75.7%)	18 (15.0%)	5 (4.2%)	4 (3.4%)	2 (1.7%)
Emotional neglect	36 (30.3%)	26 (21.3%)	42 (34.6%)	8 (6.5%)	9 (7.3%)
Physical neglect	68 (55.9%)	23 (18.9%)	13 (10.7%)	6 (5.5%)	11 (9.0%)

Values represent numbers of participants (N) and percentage of the entire sample.

### Childhood Adversity and Percent Methylation

**Figure 3:**
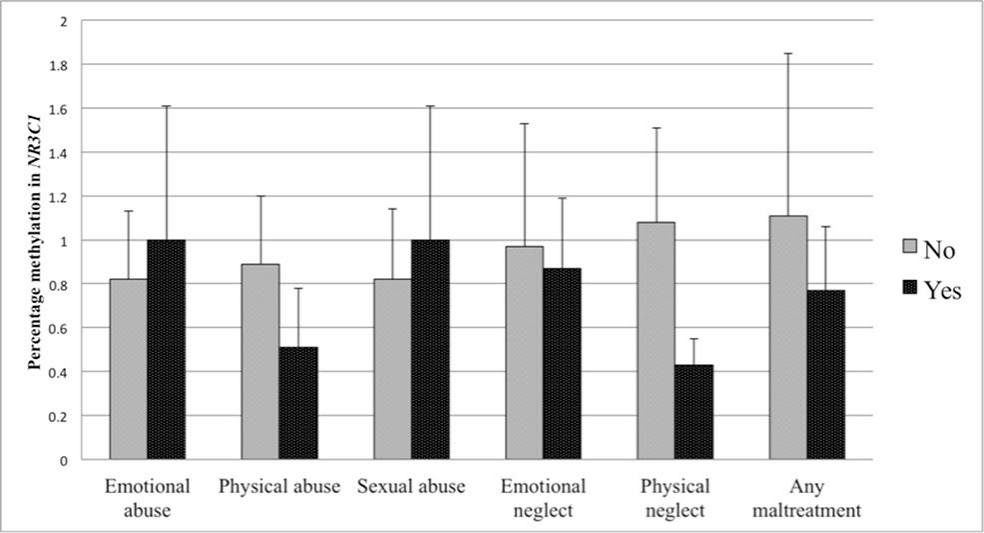
Percent methylation in the glucocorticoid receptor (*GR*) gene promoter (*NR3C1*) in women with experience of specific sub-forms of abuse and neglect (black bars) compared to women without such experiences (grey bars). Bars represent mean values in *NR3C1* methylation and whiskers represent standard errors.

**Figure 4:**
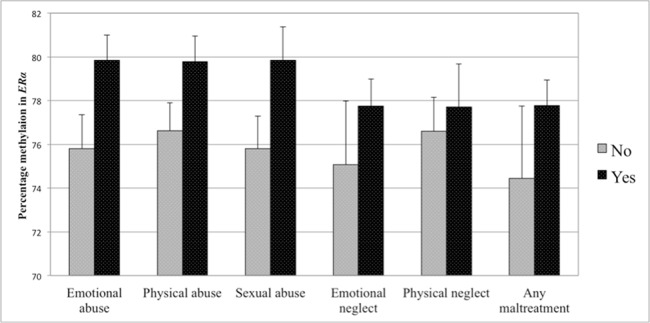
Percent methylation in the estrogen receptor alpha gene shore (*ERα*) in women with experience of specific sub-forms of abuse and neglect (black bars) compared to women without such experiences (gray bars). Bars represent mean values in *ERα* shore methylation and whiskers represent standard errors.

The first set of analyses tested the association of sub-forms of childhood adversity with methylation. There was no association of any childhood adversity sub-form with methylation in the *NR3C1* promoter when controlling for age, BMI, household income, and smoking status (see **Table 4**). Controlling for the same variables, methylation in the *ER*α shore was, however, significantly positively associated with physical neglect. The associations between different sub-forms of adversity and methylation are depicted in **Figure 3** for the *NR3C1* promoter and **Figure 4** for the *ER*α shore, revealing differences in methylation scores as a function of the experience *vs*. non-experience of the different sub-forms of childhood adversity.

**Table 4:** Associations between Childhood Trauma Questionnaire sub-forms and percent methylation.

	1	2	3	4	5	6	7	8
Log *NR3C1* promoter (1)	1.00	.07	−.08	−.03	.06	−.04	−.02	−.02
*ER*α shore (2)		1.00	.16	.18	.05	.11	.24*	.28**
Emotional abuse (3)			1.00	.75***	.21	.66***	.63***	.77***
Physical abuse (4)				1.00	.24*	.43***	.69***	.58***
Sexual abuse (5)					1.00	.19	.20	.30**
Emotional neglect (6)						1.00	.49***	.78***
Physical neglect (7)							1.00	.66***
Maltreatment score (8)								1.00

Statistics represent partial correlations (r) controlled for age, body mass index (kg/m2), annual household income, and smoking status. p < .029 = * (adjusted for multiple testing), p < .01 = **, p < .001***.

In a next step, regression analyses were performed to test whether the experience compared to non-experience of childhood adversity (dummy-coded sub-forms) was predictive for the methylation in the *NR3C1* promoter. In a first step, control variables were accounted for (*R* *^2^* = .04), while adding any of the childhood adversity sub-forms did not significantly improve the model fit (all *p* > .10). Hence, differences in *NR3C1* promoter methylation could not be explained by childhood adversity.

The same set of regression analyses was repeated with methylation in the *ERα* shore as dependent variable. After taking the control variables into account in a first step (*R* *^2^* = 13.0), the results revealed that adding any kind of maltreatment (*ΔR* *^2^* = .037, *p* = .030), emotional abuse (*ΔR* *^2^* = .089, *p* = .001), physical abuse (*ΔR* *^2^* = .027, *p* = .067), sexual abuse (*ΔR* *^2^* = .019, *p* = .106), or emotional neglect (*ΔR* *^2^* = .036, *p* = .040) improved the model fit. After controlling for multiple testing, only emotional abuse was retained as a statistically significant predictor of methylation in the *ER*α shore (see **Table 5**).

**Table 5:** Linear regression analyses with *ER*α shore methylation as dependent variable and type and severity of childhood adversity as independent variables.

		
Type of adversity		
Any childhood adversity	.20	.030
Emotional abuse	.32	.001
Physical abuse	.17	.067
Sexual abuse	.15	.106
Emotional neglect	.20	.040
Physical neglect	.11	.251
Severity of adversity		
Maltreatment score	.33	.001

Values on type of adversity represent dummy-coded variables for experience of this type of adversity (non-experience represents the reference categories). Analyses are adjusted for age, BMI, annual household income, and smoking status. p < .029 is considered as statistically significant (adjusted for multiple testing).

### Additive Effects of Childhood Adversities on Percent Methylation

**Figure 5:**
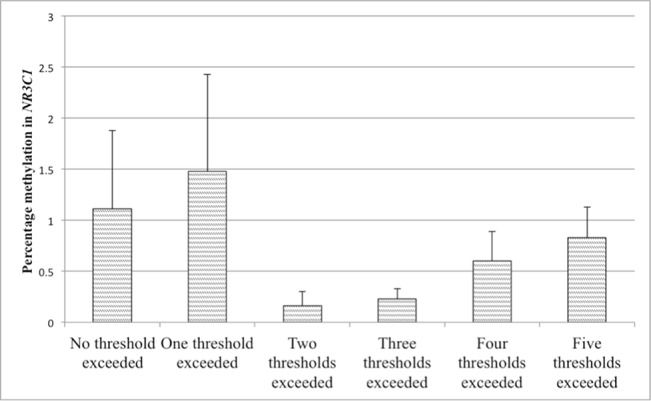
Values represent means and standard errors (SEM) of percent methylation in the glucocorticoid receptor (GR) gene promoter (*NR3C1*). A higher maltreatment score reflects a higher number of categories of abuse or neglect above a critical threshold.

**Figure 6:**
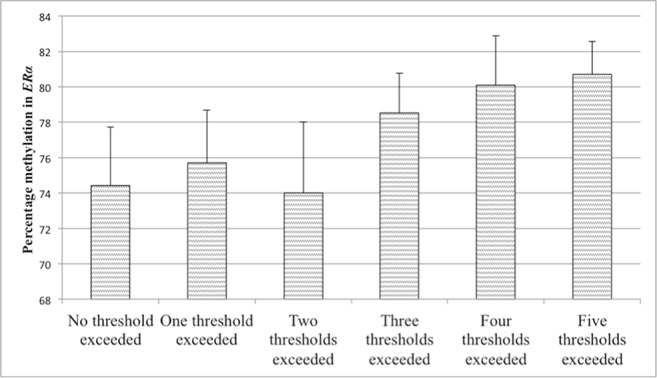
Values represent means and standard errors (SEM) of percent methylation in the estrogen receptor alpha gene shore (*ER*α). A higher maltreatment score reflects a higher number of categories of abuse or neglect above a critical threshold.

Partial correlations revealed that methylation in the *NR3C1* promoter was not associated with the score for reported maltreatment when controlling for age, BMI, annual household income, and smoking status. This lack of association was additionally confirmed in linear regression analyses. In a first step, control variables were accounted for (*R* *^2^* = .04), while adding the maltreatment score did not significantly improve the model fit (*p* > .10). The level of maltreatment was therefore not predictive for methylation in the *NR3C1* promoter. The maltreatment score was, however, significantly positively associated with methylation in the *ERα* shore. The second regression analysis therefore tested whether the maltreatment score was predictive for the methylation level in the *ER*α shore. After taking the control variables into account in a first step (*R* ^2^ = 13.0), the results revealed that adding the maltreatment score significantly improved the model fit (*ΔR* *^2^* = .094, *p* = .001). A higher maltreatment score was associated with higher *ER*α shore methylation, even when controlling for multiple testing (see **Table 5**). The association between the maltreatment score and methylation levels is illustrated in **Figure 5** for the *NR3C1* promoter and **Figure 6** for the *ER*α shore.

### Percent Methylation and Steroid Hormone Levels

Finally, the association between methylation and circulating steroid hormone levels was investigated. Methylation in the *NR3C1* promoter was negatively associated with E2 and the E2/C ratio, although these associations did not reach statistical significance after adjusting for the control variables age, BMI, annual household income, smoking status, and acute stress (*p* < .10). Methylation in the *ER*α shore was significantly positively associated with E2 and positively, although not significantly, associated with the E2/C ratio (*p* < .10; see **Table 6**).

**Table 6:** Associations between percent methylation and steroid hormone levels.

	1	2	3	4	5	6
Log *NR3C1* promoter (1)	1.00	.11	−.17	−.09	.10	−.17
*ER*α shore (2)		1.00	.21*	.10	−.02	.17
Log Estradiol (3)			1.00	.14	−.01	.78***
Log Walking Cortisol (4)				1.00	−.11	.12*
Log 8:00 am Cortisol (5)					1.00	−.64***
Log Estradiol/ Cortisol ratio (6)						1.00

Statistics represent partial correlations (r) controlled for age, body mass index (kg/m2), annual household income, and smoking status, and acute stress. p < .05 = *, p < .001***.

## Discussion

In this study, the effects of type and strength of early life adversity on the *NR3C1* promoter and the *ER*α shore methylation and subsequent steroid hormone levels were investigated in a sample of healthy middle-aged to older women. A large proportion of the sample (60%) reported emotional neglect of at least minimal intensity, with more than 40% reporting moderate or severe levels. Almost 40% reported at least minimal physical neglect, while rates for emotional abuse, sexual abuse, and physical abuse were considerably lower. As expected for a community-dwelling sample, prevalence rates for severe forms of adversity were rather low (70). Moreover, sub-forms of abuse and neglect showed high intercorrelations. Contrary to our initial assumptions, methylation in the *NR3C1* promoter was neither associated with the type nor with the strength of adversity. As proposed, the experience of adversity was in turn associated with higher methylation in the *ERα* shore, with indications of a dose-response relationship. Furthermore, higher methylation in the *ER*α shore was associated with higher E2 levels and a higher E2/C ratio. Finally, higher *NR3C1* promoter methylation was associated with higher basal cortisol levels and a lower E2/C ratio. All women in our study considered themselves as healthy, independent of a history of childhood adversity. This fact needs to be considered when interpreting the findings.

At first glance, the absence of an association between *NR3C1* promoter methylation and early life adversity seems to contradict the existing literature. Since the initial animal-based study by Weaver et al. (71), many human studies have been able to replicate a hypermethylation in *NR3C1* in children and adults with diverse pathological states who had experienced childhood adversity (i.e., 21, 26, 27 for reviews). These findings appear to be in contrast to the existing research on *NR3C1* methylation in healthy adults with a history of childhood adversity, as well as the findings of the present study, which might be attributable to methodological differences in the investigated CpG sites. Tyrka et al. (33) investigated 13 different CpGs from whole blood samples encompassing exon 1_F_. The authors reported higher methylation after maltreatment for men and women, but only in one of the investigated CpG sites, which is known to represent a gene-regulatory site (72). Shields et al. (32) reported higher methylation in *NR3C1* in women who had suffered childhood abuse compared to non-abused women. The samples in their study were analyzed from whole blood samples, and the CpG island shore located downstream of the proximal promoter region in *NR3C1* was targeted. The most recent study, by Alexander et al. (34), investigated whole blood samples in childhood trauma survivors from the same 39 CpGs in exon 1_F_ as in our study. None of the CpGs was directly associated with early life adversity. However, methylation in CpG_12_ (in line with CpG_12_ in our study) moderated the association between childhood adversity and the cortisol response to a psychosocial stress test. Individuals with higher CpG methylation showed a higher cortisol response than those with low methylation. This effect was not visible in participants without childhood trauma, independent of CpG_12_ methylation. In our study, mean % methylation levels across all 39 CpGs within exon 1_F_ were investigated in line with Palma-Gudiel et al. (21). Overall methylation across the single CpGs was not associated with childhood adversity. Correlation analyses between single CpGs and the maltreatment score did reveal some significant associations (see **Supplementary Table 2**), which need further investigation possibly in a larger sample size. Higher overall methylation was, moreover, related to higher basal cortisol levels and a less favorable E2/C ratio, although these associations did not reach statistical significance when adjusting for control variables. It can only be suspected that these markers might be indicators of low-grade HPA axis dysfunction. Stress induction using the DEX/CRH test or the Trier Social Stress Test would have provided further insights into whether *NR3C1* promoter methylation was associated with alterations in HPA axis functioning in our healthy sample. Additionally, it needs to be considered that only one of the previous studies also exclusively investigated women (32), while the others included equal numbers of men and women (33, 34). Sex differences should be taken into account when investigating NR3C1 methylation and its effect on HPA axis functioning. Additional explanations such as genetic polymorphisms of genes of interest (31), or individual differences in enzymatic activity of methlytransferase, acting on CpG substrates (73) might explain the discrepant findings among studies in healthy adults.

Our findings point in the direction of a dose-dependent relationship between childhood adversity and methylation in the *ERα* shore in women. Animal research has already revealed that early social experience can affect brain, behavior, and stress reactivities through its effect on *ERα* methylation and expression (48–50). Women are generally at a higher risk of stress-related disorders than men (8, 9), and a history of childhood adversity may potentiate this effect. Our study proposes DNA methylation in the *ERα* shore as one possible mechanism linking childhood adversity and risk of psychopathology in women. Notably, we found the association between early life adversity and the overall *ERα* shore methylation as well as for some single CpGs (see **Supplementary Table 3**) in a non-clinical sample of healthy adult women. It may be speculated that this effect could be even stronger in clinical populations and pathologies such as depression, which is thought to be linked to both estrogen actions and early life adversity (3, 74, 75).

The major strength of our study is the strict inclusion of healthy women. With this approach, we were able to rule out the effect of current psychiatric disorders on methylation and therefore investigate a “clean” sample. Only 11 participants had experienced any lifetime psychiatric disorders (mainly depression or eating disorders), and these participants did not differ from the rest of the sample either in terms of methylation or adversity (results presented in **Supplementary Table 1**).

Nevertheless, there are some methodological limitations which need to be considered when interpreting the findings of our study. Early life adversity was captured with the Childhood Trauma Questionnaire. Despite being one of the most widely used questionnaires in this field, the CTQ does not consider other early life stressors such as early parental death, or prenatal stressors such as mothers’ psychopathology, which could have had an additional effect on the epigenetic mark (26). Moreover, the experience of early life adversity was assessed through retrospective self-report, which might have been biased by social desirability. Previous studies in humans mainly assessed methylation from peripheral whole blood samples, although cell composition might pose a potential confounder and methylation profiles from peripheral cells might not adequately represent the brain state (76). From their review of the animal and human literature, Turecki and Meaney conclude that strong stressors might lead to an adaptation of the epigenome in the brain and even in peripheral cells. As such, easily accessible tissue samples from peripheral blood can be considered as a valid method to assess methylation profiles (26). We used the DBS technique, which is a relatively new sampling method in the context of methylation analyses. The latest publications are promising and suggest that this handy and simple technique is reliable and valid in the assessment of methylation marks (reviewed in 54, 63). As mentioned above, cell composition can be a potential confounder, which was not controlled for in the present analyses and therefore needs to be considered as a limitation. We excluded some biomarker values, because we considered the levels as implausibly high. Although we based these decisions on criteria such as age or menopausal stage and compared levels with findings from previous studies, the exclusion of these values still has to be considered as a possible limitation. Finally, we investigated basal profiles of steroid hormone levels. As discussed above, markers of stress reactivity might provide additional valuable insights into the mechanisms underlying disease and resilience.

To conclude, a large number of high-quality studies suggest a link between early life adversity and the risk of psychopathology later in life. The precise underlying biological mechanisms are, however, still a subject of study. For the purpose of comparability, future studies, using sex-specific analyses, are encouraged to consider the mediating role of single CpG and overall *NR3C1* promoter and *ER*α shore methylation in the association of early life adversity with both basal hormone secretion and endocrine stress reactivity in men and women. The finding regarding *ERα* shore and early life adversity needs to be replicated in further studies employing larger sample sizes, women at diverse developmental stages, and clear indicators of the source and strength of adversity. In particular, studies in children or adolescent girls could be crucial, as this would enable the effects of further lifetime stressors on the epigenetic mark to be ruled out or controlled for. Only in this way can other pathways of action, which might provide a differential or additional explanation for increased *ERα* shore methylation, be ruled out. If replicated, this mechanism of action might provide more insights into specific pathways linking early life adversity and disease, especially in women. In conclusion, rather than one single biological mechanism, a complex interplay of characteristics of exposure, sex, and biological resources in the form of genetics and epigenetic marks, with subsequent consequences for stress adaptation, seems to meditate the effect of early life adversity on risk or resilience.

## Data Availability Statement

The datasets for this manuscript are not publicly available due to data privacy regulations. Requests should be directed to s.fiacco@psychologie.uzh.ch.

## Ethics Statement

The study was conducted in accordance with the recommendations of the Cantonal Ethics Committee (KEK) Zurich, which classified the protocol as uncritical. The patients/participants provided their written informed consent to participate in this study.

## Author Contributions

UE, SF, EG, LM, and LS contributed to the conception and design of the study. SF, LM, and LS collected data. EG performed methylation analyses. SF performed the statistical analyses; all authors contributed to data interpretation. SF wrote the first draft of the manuscript. EG wrote the methods sections on methylation. All authors contributed to manuscript revision and have read and approved the submitted version.

## Funding

The University Research Priority Program (URPP) Dynamics of Healthy Aging of the University of Zurich funded the study.

## Conflict of Interest

The authors declare that the research was conducted in the absence of any commercial or financial relationships that could be construed as a potential conflict of interest.
