# Supplementary material for: DNA Methylation in Healthy Older Adults With a History of Childhood Adversity—Findings From the Women 40+ Healthy Aging Study
Source: Front Psychiatry. 2019 Oct 23;10:777. doi: 10.3389/fpsyt.2019.00777 (PMC6819958; doi:10.3389/fpsyt.2019.00777)
Supplement: Supplementary file 1 [file Table_1.pdf]

Supplement

Supplement table 1.

Comparison between women with vs. without any lifetime psychiatric disorders with respect to early life adversity sub-types and methylation levels in the *NR3C1* promoter and the *ERα* shore.

	**With lifetime psychiatric disorder**	**Without lifetime psychiatric disorder**	**U**	**Z**	**p**
Emotional abuse			383.00	-1.272	.204
Mean rank	68.20	54.79			
Sum of rank	682.00	5534.00			
Physical abuse			545.00	-.375	.708
Mean rank	55.55	58.81			
Sum of rank	611.00	6175.00			
Sexual abuse			505.50	-1.161	.428
Mean rank	51.95	58.64			
Sum of rank	571.50	6098.00			
Emotional neglect			438.00	-1.161	.246
Mean rank	67.18	55.34			
Sum of rank	739.00	5589.00			
Physical neglect			389.50	-1.601	.109
Mean rank	41.41	57.07			
Sum of rank	455.50	5649.50			
Maltreatment score			426.00	.696	.696
Mean rank	54.90	51.13			
Sum of rank	549.00	4704.00			
NR3C1 promoter methylation			442.00	-.353	.724
Mean rank	46.18	49.36			
Sum of rank	508.00	4245.00			
ERá shore methylation			504.00	.549	.549
Mean rank	63.18	56.89			
Sum of rank	695.00	5860.00			

Note. Grouping variable is lifetime psychiatric disease. *p < 0.05 uncorrected for multiple comparison; **p < 0.01, uncorrected for multiple comparison.

Supplement table 2.

Correlation analyses of single CpGs in the *NR3C1* promoter region and the maltreatment score.

	Maltreatment score	
**Site**	**r**	***p***
CpG1	**.181***	**.043**
CpG2	**.227***	**.015**
CpG3	.172	.051
CpG4	-.121	.127
CpG5	-.063	.278
CpG6	-.114	.142
CpG7	-.007	.474
CpG8	.021	.421
CpG9	-.141	.091
CpG10	-.141	.091
CpG11	-.148	.080
CpG12	-.144	.087
CpG13	-.155	.072
CpG14	-.133	.104
CpG15	-.144	.086
CpG16	-.145	.086
CpG17	-.118	.132
CpG18	-.142	.090
**CpG19**	**-.204***	**.026**
CpG20	-.140	.093
CpG21	-.139	.094
CpG22	-.139	.094
CpG23	-.135	.100
**CpG24**	**-.182***	**.043**
CpG25	-.143	.088
CpG26	-.135	.101
CpG27	-.119	.130
CpG28	-.116	.136
CpG29	-.145	.086
CpG30	-.141	.091
CpG31	-.136	.100

CpG32	-.092	.193
CpG33	-.103	.166
CpG34	-.080	.225
CpG35	-.102	.169
CpG36	-.049	.322
CpG37	.126	.117
CpG38	-.104	.164
CpG39	-.054	.307

Note. *p < 0.05 uncorrected for multiple comparison; **p < 0.01, uncorrected for multiple comparison.

Supplement table 3.

Correlation analyses of single CpGs in the *ERα* shore and the maltreatment score.

	**Maltreatment score**	
**Site**	**r**	***p***
CpG1	**.215***	**.017**
CpG2	.141	.085
CpG3	.084	.205
CpG4	.098	.170
CpG5	.167	.051
CpG6	.092	.186
CpG7	.165	.054
CpG8	.106	.151
CpG9	.049	.318

Note. *p < 0.05 uncorrected for multiple comparison; **p < 0.01, uncorrected for multiple comparison.
